# Robotic radiosurgery versus micro-multileaf collimator: a dosimetric comparison for large or critically located arteriovenous malformations

**DOI:** 10.1186/1748-717X-8-205

**Published:** 2013-08-23

**Authors:** Sławomir Blamek, Aleksandra Grządziel, Leszek Miszczyk

**Affiliations:** 1 Department of Radiotherapy, Maria Skłodowska-Curie Memorial Cancer Center and Institute of Oncology, Gliwice Branch, Gliwice, Poland; 2 Department of Radiotherapy and Brachytherapy Planning, Maria Skłodowska-Curie Memorial Cancer Center and Institute of Oncology, Gliwice Branch, ul. Wybrzeże AK 15, 44-100, Gliwice, Poland

**Keywords:** Arteriovenous malformations, Dose distribution, CyberKnife, Linear accelerator, Micro-multileaf collimator

## Abstract

**Background:**

Stereotactic irradiation of large or critically located arteriovenous malformations (AVMs) is a special challenge for clinicians and radiation physicists. To date, no comprehensive comparison of two linac-based radiosurgery systems used for hypofractionated radiotherapy of large AVMs was published. The aim of the study was to compare dose distributions between CyberKnife (CK) system and linac with a micro-multileaf collimator (L-mMLC) in high-grade or critically located cerebral AVMs.

**Methods:**

Two sets of plans made for 15 different patients with at least 95% target coverage were selected for comparisons. Conformity (CI), homogeneity (HI) and gradient score (GSI) indices, conformity index proposed by Lomax (CIL), conformation number (CN), quality of coverage (Q), volumes of brain receiving 12,10,8,6,4, and 2 Gy, minimum and maximum doses for critical structures in both treatment planning systems (TPS) were compared. Finally, the number of monitor units needed to deliver the prescribed dose was compared.

**Results:**

The mean minimum doses in the target volume were 93.3% (CK) and 90.7% (L-mMLC),p=n.s, maximum: 119.7 and 110%, respectively (p=0.004). The mean CI was 1.46 and 1.86, HI: 1.2, and 1.11, CIL 0.7, and 0.6, CN: 0.68 and 0.58 for CK and mMLC, respectively (p<0.05). The values of GSI and Q were not significantly different. The volumes of the brain receiving low doses (4 Gy and 2 Gy) were significantly lower in the CK system. The number of monitor units necessary to deliver the prescribed dose was significantly greater in case of the CK system.

**Conclusions:**

Better conformity can favor the CK system for treatment of large AVMs at the cost of higher maximum doses and worse homogeneity. L-mMLC is superior when shorter treatment time is required. Neither system can assure satisfying dose gradients outside large targets surrounded by numerous critical structures.

## Background

Large and critically located arteriovenous malformations are consistently a challenge for clinicians. Traditionally a “wait and see” policy was proposed to patients harboring AVMs unsuitable for surgery, efficient endovascular treatment or stereotactic radiosurgery. Recent literature indicates however, that large size of an AVM can be an independent risk factor of bleeding which stands in contrast to conclusions drawn from the earlier studies [1]. Moreover, a large populational study conducted by the Finnish group provided evidence that active, even partial treatment may be of benefit for patients with cerebral AVMs [2]. Better understanding of the natural and altered by the treatment course of the disease results with growing interest in treatment of large-volume lesions. Advances in microneurosurgery allow for removal of previously inaccessible lesions and patients unfit for surgery are more often qualified for stereotactic radiotherapy. AVMs involving critical structures can be treated with hypofractionated stereotactic radiotherapy (HFSRT), whereas lesions of large volume are suitable both to volume-staged radiosurgery and HFSRT [3,4].

Gamma knife, linear accelerators and heavy ion irradiation have a long history of use for treatment of arteriovenous malformations with gamma knife being the first device used for stereotactic radiosurgery for cerebral AVMs [5-8]. Along with development of linear accelerators both specialized accessories like micro-multileaf collimators and dedicated units for radiosurgery become available and widely used. The micro-multileaf collimator (mMLC) allows for field shaping and intensity modulation which is not possible in case of gamma knife system. As a next step of development of linear accelerators, the CyberKnife (CK) system was built. Its capabilities resemble those characteristic for gamma knife. Additionally, it does not require invasive fixation of the head because of continuous tracking and correction for intrafraction movements. Although the most recent model of the gamma knife device – GammaKnife Perfexion can also be used for fractionated treatment with immobilization with a mask, still a vast number of gamma knife centers utilize the older models. The CyberKnife system is currently used for treatment of various conditions like primary or metastatic malignant neoplasms and non-neoplastic lesions including arteriovenous malformations. The published results of treatment are uncommon but resemble that obtained with other radiosurgery systems [9,10]. The literature dealing with dose distribution generated by the CyberKnife system in radiosurgery for AVMs in relation to other radiosurgery is even more scarce [3,11]. To date, no comparisons between CK and linear accelerator with micro-multileaf collimator (L-mMLC) were made for large-volume lesions treated with hypofractionated stereotactic radiotherapy.

The aim of the current study was to compare dose distributions obtained in the CyberKnife system and in the system composed of a linear accelerator with a mMLC for large, involving, surrounding or abutting critical structures arteriovenous malformations.

## Methods

### Patients

**Figure 1:**
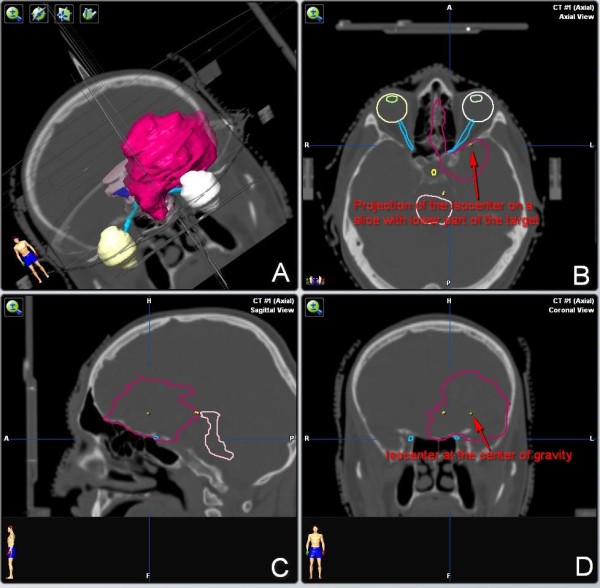
**An example of a complex shape of an AVM. A** – 3D representation of the target volume. AVM has a complicated shape and is located next to the brainstem and encompasses the left optic nerve. **B** – transverse view of the lower part of the AVM, projection of the isocenter is near the edge of the target volume, radii measured from this point would be significantly different from those measured at the level of the isocenter. **C** – sagittal view of the AVM at the level of the isocenter. The shape at this level appears to be quite regular. The target volume is adjacent to the brainstem. **D** – coronal view of the AVM, irregular shape of the AVM is visible better than on the sagittal projection.

**Figure 2:**
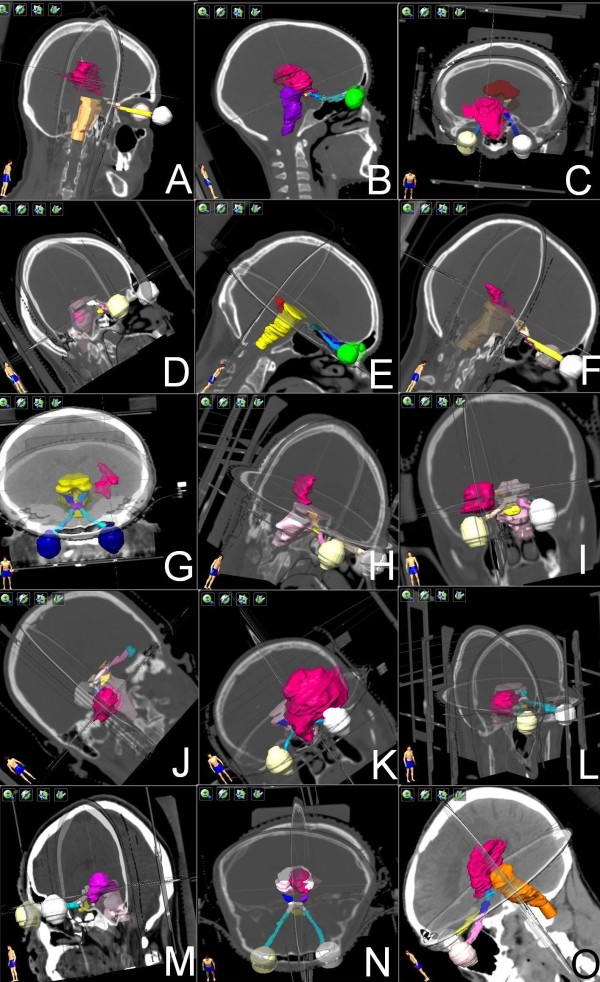
**Three-dimensional views of the target volumes. A** – case 1 – large AVM close to the optic chiasm and brainstem. **B** – case 2 - large AVM involving brainstem and close to the optic pathway. **C** – case 3 - large AVM encompassing the right optic nerve and close to the brainstem, optic chiasm and right optic tract. **D** – case 4 - small AVM located inside the brainstem. **E** – case 5 – small AVM next to the brainstem, **F** – case 6 – irregular AVM partially located inside the brainstem, **G** – case 7 – irregular AVM next to the brainstem, **H** – case 8 – AVM next to the brainstem, **I** – case 9 – AVM adjacent to the right optic nerve, **J** – case 10 - AVM involving the brainstem, **K** – case 11 – large AVM encompassing the left optic nerve and adjacent to the optic chiasm and brainstem, **L** – case 12 – AVM adjacent to the right optic tract and close to the optic chiasm, **M** – case 13 – AVM involving the brainstem and adjacent to the optic chiasm, **N** – case 14 - AVM inside the brainstem and adjacent to the left optic tract, **O** – case 15 – large AVM adjacent to the brainstem and optic chiasm.

15 patients with high-grade or critically located AVMs were selected and included into the study. The mean volume of the lesion was 21.7 cm^3^ and ranged from 1.02 to 146.45 cm^3^. There were 11 Spetzler-Martin grade III, 3 grade IV, and 1 grade V AVMs. All lesions were located in close proximity or involved critical structures like brainstem or optic pathway. Due to complicated shape of the AVMs there was no reliable metrics to be used to describe the lesions. As there is no proper data in treatment planning systems (for instance the surface of the target) to calculate the sphericity value, it could not be used to describe the shape of the PTVs. Potential approach to roughly estimate the surface of the PTV would in our opinion lead to obtaining values with error significantly larger than the accuracy the other values were measured and thus, it would lead to unreliable results of comparisons. The measure of sphericity determined by measuring three linear dimensions is in our opinion an insufficient method to describe shapes of complicated 3D objects. The diameters vary significantly with the subsequent CT scans (Figure 1). Therefore, definition of the matrix of the diameters would be required. For the same reasons we rejected the idea to define the sphericity on the basis of the radius measured from the center of gravity of the target. To give an idea of the complexity and location of the target volumes and of steepness of dose fall-off necessary to preserve the critical structures we have chosen to present the three-dimensional reconstructions of the target volumes to depict their complexity and relation to organs at risk (Figure 2). Moreover, detailed description of the AVMs with their volumes and relation to OARs is presented in the Table 1.

**Table 1:** Detailed characteristics of arteriovenous malformations included into the study with respect to their volume and relation to the nearest organ at risk

**Case**	**Volume (cm**^**3**^**)**	**Distance (mm)**	**Nearest OAR**	**Relation to OAR**
1	29.30	9.4	chiasm	neighboring
2	24.67	0	brainstem	involving
3	22.93	0	optic nerve R	encompassing
4	1.02	0	brainstem	within
5	1.14	1	brainstem	neighboring
6	4.72	0	brainstem	involving
7	3.17	1.3	brainstem	neighboring
8	5.62	1	brainstem	neighboring
9	11.78	2.2	optic nerve R	neighboring
10	13.66	0	brainstem	involving
11	146.45	0	optic nerve L	encompassing
12	17.76	0	optic tract R	adjacent
13	9.05	0	brainstem, chiasm	Involving brainstem, adjacent to chiasm
14	6.42	0	brainstem	within
15	27.83	0	brainstem	adjacent

L -left, R – right, neighboring – the PTV is located at some distance from the nearest OAR, involving – the PTV is partially inside the nearest OAR, encompassing – the PTV surrounds the nearest OAR, adjacent – the PTV is in contact with the nearest OAR but does not surround or involve it, within – the whole PTV is inside an OAR.

Radiosurgery-based AVM score was also calculated for each lesion and had a mean value of 3.13 (range: 1.07 to 15.41). Radiosurgery-based AVM score is calculated according to the following formula: AVM score = 0.1 volume (mL) + 0.02 age (years) + 0.3*location, where location is a two-tiered variable depending on the location of the AVM: for hemispheric, corpus callosum and cerebellar location it has the value of 0, whereas for AVMs located in basal ganglia, thalamus and brainstem it is equal to 1 [12]. AVM score allows for prediction of treatment outcome and the greater the value the smaller probability of obliteration after stereotactic irradiation [12].

#### Imaging, target definition and dose prescription

All the imaging data were imported into the MultiPlan treatment planning system (TPS) developed for the CyberKnife system. Radiosurgery planning was based on computed tomography (CT), post-contrast T1 and FLAIR magnetic resonance images (MRI), and magnetic resonance angiography (MRA). Image fusion was performed using an automated intensity-based algorithm that maximizes the mutual information of the loaded images, and corrected manually when necessary. The target volume and critical organs (brainstem, optic nerves, optic chiasm, optic tracts, pituitary gland, eyeballs, lenses, and uninvolved brain) were delineated in the MultiPlan system and subsequently transferred to iPlan TPS without modifications. The target volume was defined as the AVM nidus without draining veins, whereas organs at risk (OARs)were outlined according to anatomy. Prescription of the dose was based on size and location of the AVM and proximity of organs at risk. In all cases hypofractionation was chosen to assure tolerable doses for organs at risk and to avoid irradiation of large volumes of the brain with high doses. According to our protocols, three fractionation schedules were used: 3 × 7 Gy, 3 × 8 Gy, and 4 × 6 Gy. All the reported total doses for the target volume are an arithmetic sum of physical fraction doses, the reported maximum and mean doses are maximum and mean values of the total dose. Similarly, the doses delivered to organs at risk are physical doses calculated for the whole treatment plan (all fractions). No calculation of biological equivalent doses was performed.

#### Treatment planning - CyberKnife

All treatment plans were prepared in the MultiPlan version 4.5.0 (Accuray Incorporated, Chesapeake Terrace Sunnyvale, CA, USA). In all cases fixed cylindrical collimators were used. We did not use the Iris collimator in order to assure maximum steepness of dose falloff resulting from narrower penumbra of fixed collimators, and to avoid dose uncertainties that can be expected when the smallest diameters of the Iris collimator are used [13]. All plans were made with nonisocentric inverse-planning technique. In all cases shells were used to assure steep dose gradient outside the target volume. The number and diameter of collimators were selected individually, according to the size and shape of the lesion. The pencil-beam algorithm was used for dose distribution calculation. The number of beams used ranged from 76 to 375 (mean and median 183, and 171, respectively). The optimization process was repeated until no further improvement regarding dose distribution and critical organs constraints could be achieved.

#### Treatment planning - linear accelerator with mMLC

Treatment plans for L-mMLC were made with iPlan version 4.1.1 (Brainlab AG, Feldkirchen, Germany) for the m3 micro-MLC (Brainlab AG, Feldkirchen, Germany) with leaf width of 3 mm at the isocenter. In all cases both intensity-modulated radiosurgery plans (IMRS) and plans with a set of fixed-angle static fields were prepared. The number of beams ranged from 9 to 15 (mean and median 12). The gantry angles and leaf positions were manually adjusted in the static-field plans to achieve the best dose distribution. IMRS plans were optimized as long as no further improvement in dose distribution could be gained. Plans with dynamic conformal arcs were also prepared. They were however not useful and were not selected for comparisons because of unacceptable dose distribution in case of large and irregularly shaped target volumes. According to our experience dynamic conformal arcs give the best results for small target of spherical or ellipsoid shape. Similar findings (better performance of dynamic conformal arcs for smaller targets) were published by Wiggenraad et al. [14]. Superiority of IMRT over dynamic conformal arcs for stereotactic irradiation of complex, concave or irregular target volumes was also demonstrated by Sharma et al. [15]. The plans were compared within the TPS and the optimal plans that in the greatest degree fulfilled the predefined criteria were chosen for further comparisons.

#### Plan selection criteria and comparisons

All plans were prepared by the same experienced radiosurgery team in working in our Center and utilizing both treatment systems (around 5000 patients treated with a linear accelerator with mMLC and about 900 with CK until now). All plans were optimized to assure at least 95% coverage of the target volume with the prescribed dose to facilitate further comparisons. Moreover, the plans had to fulfill the predefined dose constraints for organs at risk as it is defined in the protocols used in our Department (Table 2). The best plans with the assumed coverage and optimal sparing of critical organs were selected for comparisons.

**Table 2:** Dose constraints used for the hypofractionated schedules of stereotactic irradiation

**Organ at risk**	**Maximum dose (Gy), three fractions regimen**	**Maximum dose (Gy), four fractions regimen**
Brainstem	7.23	6.5
Optic chiasm	6.5	6
Optic nerves	6.5	6
Optic tracts	6.5	6
Lenses	0.5	0.5
Eyeballs	2	2
Pituitary	6	5.5

The plans were evaluated by analysis and comparison of dose-volume histograms (DVH) for AVMs and organs at risk, target coverage, and two indices proposed by the Radiation Therapy Oncology Group (RTOG) do describe the quality of stereotactic radiosurgery plans: conformity index (CI), and homogeneity index (HI) [16]. CI is defined as a ratio of total volume covered by reference isodose and the target volume, whereas HI is defined as a ratio of maximum dose in the target volume and the prescribed dose. Moreover, quality of coverage (Q), conformity index proposed for evaluation of radiosurgery treatment plans by Lomax and Scheid (CI_L_), conformation number (CN) and gradient score index (GSI) were calculated for each plan.

Quality of coverage is a ratio of the minimum dose delivered to the target volume and the prescription isodose [16]. CI, HI, and Q indices were analyzed according to the criteria proposed by RTOG to evaluate the quality of radiosurgery [16]. CI_L_ is defined as a ratio of the volume within the target irradiated with at least the prescription dose and the total volume enclosed by the prescription isodose [17]. Conformation number was originally introduced to quantitatively assess the degree of conformality in brachytherapy and can be treated as a measure of the quality of the plan [18]. It is defined as follows:

$$\mathrm{CN}={\mathrm{TV}}_{\mathrm{RI}}/\mathrm{TV}*{\mathrm{TV}}_{\mathrm{RI}}/V_{\mathrm{RI}},$$

where TV_RI_ is the target volume covered by the reference isodose, TV is the target volume, and V_RI_ is the volume of the reference isodose. Gradient score index (GSI) is a measure of the steepness of the dose falloff outside the target volume [19]. It is calculated as follows:

$$\mathrm{GSI}=100-100*\left[\left(R_{\mathrm{Eff}50\%\mathrm{IDV}}-R_{\mathrm{EffPTV}}\right)-0.3\right]$$

where R_Eff50%IDV_ is the effective radius of the 50% isodose volume, whereas R_EffPTV_ is the effective radius of the target volume assuming that both volumes are spheres [19].

To facilitate comparisons, improvement ratios for each evaluated parameter were calculated and expressed as a percentage of improvement or deterioration (Δ HI, Δ CI, Δ CI_L_, Δ Q, Δ CN, and Δ GSI). Improvement ratios were defined as follows:

$$\Delta \;\mathrm{EP}=\left({\mathrm{EP}}_{\mathrm{CK}}{‒\mathrm{EP}}_{L‒\mathrm{mMLC}}\right)/{\mathrm{EP}}_{L‒\mathrm{mMLC}}*100\%$$

where Δ EP is the improvement ratio of the evaluated parameter, EP_CK_ is the evaluated parameter calculated for CK, and EP_L-mMLC_ is the evaluated parameter calculated for L-mMLC.

Another step of plan evaluation was calculation and comparison of low, medium, and high isodose volumes (IDV) of 2, 4, 8, 10, and 12 Gy (IDV_2Gy_, IDV_4Gy_, IDV_8Gy_, IDV_10Gy_, and IDV_12Gy_, respectively) for each plan. The isodose volumes were represented both by absolute values (in cm^3^) and percentage of total tissue volume (IDV_2Gy%_, IDV_4Gy%_, IDV_8Gy%_, IDV_10Gy%_, and IDV_12Gy%_, respectively).

Moreover, three volume ratios were calculated to additionally assess the steepness of the dose falloff outside the target volume. The first one was the ratio of 80% IDV to PTV, then 60% IDV/PTV and 40% IDV/PTV. Comparison of the volumes receiving 80%, 60% and 40% of the dose to the volume of the target allows for quantitative assessment of the dose gradient. The smaller the ratios the steeper is the dose gradient [20].

As the final step, the number of monitor units necessary to deliver the demanded dose was compared. It can be considered a surrogate of the time needed to complete the treatment. Whereas the MultiPlan TPS calculates the estimated treatment time per fraction (ETTPF), similar information is not available in the iPlan TPS which was the reason to use the number of monitor units for comparisons. The research was in compliance with recommendations of the Helsinki Declaration (1964, with later amendments). Approval of the institutional Ethics Committee is not required in our Institution for a dosimetric study performed on existing imaging data.

All statistical comparisons were made with Student’s *t*-test for dependent samples. The differences were considered statistically significant if p values were < 0.05. All the statistical calculations were made with the Statistica 7.0PL software.

## Results

### Dose distribution

In all cases the 95% coverage of the target volume could be obtained. The mean coverage for the CK and L-mMLC was 97.9% and 96.7%, respectively, and the difference was not significant. In three plans made for L-mMLC and in one for the CK system (point maximum dose for brainstem of 28.57 Gy in the four-fractions regimen), the dose constraints for critical structures were violated. The minimum doses for the target volumes were comparable (mean 93.3% and 90.7%, for the CK and L-mMLC, respectively), whereas the maximum doses were significantly larger in the CK system (mean 119.7%) than in case of L-mMLC (mean 110%), p<0.05. The mean CI was 1.48 and 1.86 for CK and L-mMLC, respectively (p<0.05). In none of the CK plans the CI was greater than 2 and thus all plans were compliant with the RTOG protocol. In contrast, three L-mMLC plans had minor deviation and one with the CI value of 2.83 had a major deviation. The difference in conformity was further confirmed by significant difference in CI_L_ between systems. Analysis of the HI revealed that all the plans could be classified as not deviating from the RTOG protocol. Nevertheless, the difference between homogeneity indices between systems was highly significant (p<0.05). The Q value was found to be below 90% in two CK plans (83% and 67%, minor and major deviation, respectively), and in four L-mMLC plans (85%, 80%, 79% and 73% - two minor and two major deviations). The subsequent IDVs were larger in the L-mMLC system. The differences between high-dose isodose volumes were not statistically significant. The low dose IDVs, however, (IDV_4Gy%_ and IDV_2Gy%_) were significantly larger in plans made for L-mMLC (Table 3) indicating that low-dose isodoses were better conformed to the target volume in the CK system.

**Table 3:** Comparison of subsequent isodose volumes between CK and L-mMLC systems

	**CK ±SD**	**L-mMLC±SD**	**P value**
IDV_12Gy_	61.77±66.58	70.04±75.15	0.09
IDV_12Gy%_	4.40±4.62	6.08±5.72	0.10
IDV_10Gy_	83.43±83.70	96.50±98.75	0.07
IDV_10Gy%_	5.96±5.8	8.26±7.35	0.08
IDV_8Gy_	118.03±105.52	137.12±135.49	0.09
IDV_8Gy%_	8.48±7.31	11.72±10.10	0.07
IDV_6Gy_	179.76±138.73	218.55±188.09	**0.04**
IDV_6Gy%_	12.90±9.66	18.44±13.79	**0.03**
IDV_4Gy_	308.33±192.27	385.71±263.30	**0.01**
IDV_4Gy%_	22.17±13.46	31.92±18.48	**<0.05**
IDV_2Gy_	583.20±264.75	608.47±316.86	0.58
IDV_2Gy%_	41.90±18.35	50.40±22.08	**0.04**

CK-CyberKnife, L-mMLC – linac with micro-multileaf collimator, SD – standard deviation.

Volumes are presented both in absolute values and as ratios to total tissue volume.

Significant differences are marked with bold font.

The improvement ratios for HI (ΔHI) ranged between −4.2 and 19.8% with the majority (12) positive values indicating better homogeneity in most L-mMLC plans. ΔCI ranged from −44 to 23.3% with the majority (13) of negative values indicating better conformity in the majority of CK plans. Mean values of the other measures and their improvement ratios are summarized in Table 4.

**Table 4:** Mean values of the evaluated indices for all plans in both systems

**System**	**HI**	**ΔHI (%)**	**CI**	**ΔCI (%)**	**CI**_**L**_	**ΔCI**_**L **_**(%)**	**GSI**	**ΔGSI (%)**	**CN**	**ΔCN (%)**	**Q**	**ΔQ (%)**
CK	1.20	8.1	1.48	−20.43	0.70	16.60	26.38	−25.30	0.68	17.24	0.93	3.33
L-mMLC	1.11		1.86		0.60		35.32		0.58		0.90	
P value	**<0.05**		**<0.05**		**<0.05**		0.26		**<0.05**		0.28	

CK-CyberKnife, L-mMLC – linac with micro-multileaf collimator, HI – homogeneity index, CI – conformity index, CI_L_ – conformity index proposed by Lomax, GSI – gradient score index, CN – conformation number, Q – quality of coverage, Δ – improvement ratio of a given measure (%).

Significant differences are marked with bold font.

The 80% IDV/PTV, 60% IDV/PTV and 40% IDV/PTV ratios were comparable between systems indicating that the dose gradient did not differ significantly between CK and L-mMLC.

### Critical organs sparing

The dose distribution within critical organs was different between systems. There were significant differences in the maximum dose in the brain with and without the target volume. Higher point maximum doses were observed in case of the CK system. The detailed analysis shows also that the mean maximum doses for organs at risk are slightly higher in case of robotic radiosurgery but the difference was significant only for one structure – the left optic nerve. The detailed comparison is shown in Table 5.

**Table 5:** Maximum doses in organs at risk

**Organ**	**Mean maximum dose (Gy) CK**	**Mean maximum dose (Gy) L-mMLC**	**P value**
Brain	27.91	25.67	**<0.05**
Uninvolved brain	27.64	25.56	**<0.05**
Brainstem	20.69	20.42	0.72
Optic chiasm	12.33	11.80	0.39
Left optic nerve	7.57	5.58	**<0.05**
Right optic nerve	8.13	6.90	0.09
Left optic tract	12.21	12.01	0.83
Right optic tract	13.56	12.98	0.45
Left eyeball	3.97	2.97	0.05
Right eyeball	3.58	2.58	0.08
Left lens	0.46	0.59	0.47
Right lens	0.54	0.46	0.61
Pituitary	10.73	9.85	0.16

Significant differences are marked with bold font.

### Treatment time and number of monitor units

The difference between the number of monitor units needed to deliver the planned dose was highly significant (p<0.05). In all cases then number of monitor units was greater for the CK system (Table 6).

**Table 6:** Number of monitor units (MU) per fraction specified for consecutive cases for the CyberKnife system (CK) and linac with micro-multileaf collimator (L-mMLC)

	**Case number**														
**System**	**1**	**2**	**3**	**4**	**5**	**6**	**7**	**8**	**9**	**10**	**11**	**12**	**13**	**14**	**15**
CK	8210	25495	9616	6852	10984	14199	16649	10838	6611	12165	10188	9969	9773	7699	9063
L-mMLC	1021	815	917	903	1057	842	1145	1140	1057	823	843	949	833	858	768

## Discussion

According to the best knowledge of the authors this is the first paper to describe the comparison of dose distribution between CK and L-mMLC for large, critically located and mostly irregularly-shaped targets characteristic for high-grade AVMs. The comparisons published to date were mainly focused on small or medium-sized and roughly spherical targets, like in case of cerebral metastases, acoustic neuroma or trigeminal neuralgia [21,22]. In the paper dealing with dosimetric comparisons of stereotactic radiosurgery realized with the GammaKnife Perfexion, CyberKnife and Novalis-Tx for cerebral AVMs, the target volumes are relatively small with the mean and median volume of 6.12 and 3.76 cm^3^, and ranging between 0.51 and 17.16 cm^3^[11]. This makes such comparisons similar to the other studies [21,22]. Foght et al. published a study on seven patients with AVMs of the mean volume of 11.3 cm^3^ and ranging between 8 and 15 cm^3^[3]. They compared only conformity and homogeneity indices and 12 Gy isodose volume between the GammaKnife Perfexion and the CyberKnife. The current study is based on an analysis of dose distribution for critically located targets of wide range of volumes with the mean volume almost twice as high as published to date and exceeding the maximum volumes of AVMs described in the comparison studies until now [3,11]. All the AVMs were located in close vicinity or involved critical structures. As selecting targets of similar (small or large) volumes could unintentionally lead to a bias favoring one system (CK was reported to better spare critical organs in case of small targets like vestibular schwannoma), a diverse set of AVMs of different volumes was chosen to assure reliable comparisons between the systems [21]. The literature dealing with dosimetric comparisons of the CyberKnife system with other treatment modalities is sparse and thus, confrontation of our results with outcomes reported by other authors is difficult because of other types of lesions described or different population of patients with AVMs included in the other studies [3,11,21,22]. As a consequence, the results of the current study give an additional information on the dependence of dose distribution and quality of stereotactic plans on volume of the lesion and its relation to critical structures which cannot be found elsewhere.

The coverage of the target was comparable in both systems being slightly higher in the CK system. The mean Q was high in both systems indicating that in most cases the nidus was covered with the desired dose without significant cold spots. Detailed analysis shows however, that the number of deviations from the RTOG protocol was twice as high in case of the L-mMLC than as for the CK system. Observed deviations resulted from the need for sparing adjacent OARs. This treatment planning objective could be obtained with better quality of coverage with the CK system in the current series.

Analysis of conformity indices demonstrated that better conformity can be obtained with the CK system. However, in both systems minor deviations from RTOG protocol were noted. In case of the L-mMLC also one major deviation was observed. Apart from the one major deviation, the rest of the plans could be considered satisfactory basing solely on the CI. The CI_L_ also indicates that conformity of the plans is significantly better in case of the CK system (higher CI_L_ values mean better conformity as opposed to CI) and are within the range reported by Lomax et al. [17]. Improvement ratios for both conformity indices are on the order of 20 and 16%, which further confirms the superiority of the CK system. Neither the optimization algorithm during the inverse planning of IMRS nor experience and expertise in beam arrangement and shaping in case of static fields could assure conformity equal to that obtained with the CyberKnife system. The better the conformity, the less uninvolved tissue is exposed to high doses of radiation which has a direct impact on the risk of radiation-induced side effects. The incidence of radiation-induced abnormalities seen in MR imaging is reported to be around 30% and the risk of occurrence of the alterations and symptomatic injury increases with increased volumes irradiated to higher doses, e.g. 12 Gy [23,24]. More detailed analysis of dose distribution, however, shows that the degree of conformity and dose gradient around target volumes are less satisfactory than expected. The mean conformation numbers between 0.6 and 0.7 indicate that the ability of sculpting the isodose distribution around the nidus is limited in both systems. This is the result of specific arrangement of the beams being the consequence of avoiding OARs and delivering the dose to large target volume at the same time. Spreading the dose among the critical structures caused lower degree of conformation and influenced also the dose gradient away from critical structures.

GSI values were below expectation in both systems. This is the result of irradiation of large target volumes surrounded by numerous critical structures. The need for sparing organs at risk requires nonuniform beam arrangement with most of the beams placed in a way allowing to avoid direct irradiation of critical structures. It allows for steep dose gradients in regions adjacent to OARs but at the cost of dose spillage between them. The resulting dose falloff in regions where beams are accumulated is far from the steep gradient next to the critical structures. The effect of accumulation of the beams is aggravated in most cases by their large diameter which results with partial overlapping of the neighboring beams. The improvement ratio calculated for GSI was relatively high, the difference between system was however not significant. This is the result of large range of obtained values in both systems being the consequence of diverse volumes of the AVMs. Choosing more homogeneous group of targets in terms of their volume could probably lead to more unequivocal results but at the cost of possible unwanted favoring of one system. The similarity of dose gradient was further confirmed by the analysis of ratios of subsequent isodose volumes to the target volume revealing lack of significant differences in all cases. It means that in both systems irradiation of volumes requiring steep dose gradients next to the critical structures is associated with wide dose gradient in regions to which dose restrictions are not applied.

The maximum doses delivered to critical organs were slightly higher mainly in the CyberKnife system. This can be the result of better dose coverage and quality of coverage obtained in the CyberKnife system and thus, slightly higher doses on the surface of the lesion abutting critical structures. Moreover, the differences were not statistically significant (apart form the left optic nerve which not necessarily is the result of superiority of one system but may be due to a chance). The observed outcomes may result from heterogeneity of the group (both large AVMs and relatively small lesions but located in the vicinity of critical structures were included) and limited number of the cases analyzed. As a consequence, the study can be underpowered to demonstrate significant differences between systems. On the other hand, the number of cases included into the current study is the largest in the literature dealing with dosimetric comparisons of stereotactic radiosurgery systems in the setting of AVM treatment [3,11]. Higher maximum doses and worse homogeneity in case of CK can potentially affect the treatment outcome. Whether the differences in quality measures observed in the current study are of clinical importance is an open question and require a clinical study. In clinical practice the plans selected for treatment would probably be those with safe doses to critical organs at the cost of slightly lower coverage of the target volume. However, for the purpose of the study we have chosen to define uniform selection criteria (i.e. 95% coverage and dose constraints for critical organs), to reliable compare the rest of measures used for evaluation of the treatment plans. The doses allowed to certain critical organs can differ between treatment protocols implemented in other centers. We have chosen the dose constraints used in our Department which can be considered relatively conservative. A potential drawback of the study can also be the assumption that all the lesions will be treated with hypofractionated stereotactic radiotherapy. It is important to note that we have selected only high-grade and critically located AVMs for the purposes of this study. Some of them were previously treated with embolization without success and in any of them we would not propose a single fraction treatment due to either large volume or critical location of the lesion or both. Hypofractionated stereotactic radiotherapy is a treatment modality used in our institution in such cases [4]. Volume-staged radiosurgery would be extremely difficult to implement in our study because between consecutive treatments some changes in the AVM shape and volume could be expected which would make the comparisons irrelevant.

The most significant difference between the systems was the number of monitor units required to deliver the planned dose. This finding is in line with dosimetric comparisons of robotic radiosurgery and linac-based systems in other locations [25,26]. Roughly tenfold higher number of monitor units needed in the CK systems leads to significant elongation of the treatment time. It can be of importance in case of patients whose medical condition does not allow for long irradiation. On the other hand, AVMs are diagnosed mainly in young and middle-aged patients which allows for less restricted constraints concerning the treatment time than for example in case of patients with disseminated neoplastic disease irradiated for cerebral metastases.

## Conclusions

Both systems allow for irradiation of complex-shaped and large target volumes surrounded by critical structures with satisfactory coverage but neither is able to generate satisfactory dose gradients outside such lesion. Better conformity of the plans is an argument in favor of choosing the CyberKnife system for irradiation of lesions close to OARs. On the other hand, better homogeneity of the plans made for linear accelerator with micro-multileaf collimator along with significantly smaller number of monitor units needed to complete the treatment can favor this system in patients unable to maintain the treatment position for long time or in cases where homogeneous dose distribution is preferred and the occurrence of hotspots is of special concern.

## Competing interests

SB - Travel grant from TMS Sp. z o.o., AG - Employed as a translator of CyberKnife manuals, travel grant, LM- Travel grant from Varian (Varian Oncology Summit - 2011), consultancy, agreement with Varian concerning educational activity.

## Authors’ contributions

SB provided design of the study, analyzed the treatment plans, performed the statistical analysis, revised the literature and prepared the manuscript, AG prepared the treatment plans and helped in their analysis and preparation of the manuscript, LM participated in coordination and helped to draft the manuscript. All authors have read and approved the final manuscript.
